# Achilles Tendinopathy: Current Concepts about the Basic Science and Clinical Treatments

**DOI:** 10.1155/2016/6492597

**Published:** 2016-11-03

**Authors:** Hong-Yun Li, Ying-Hui Hua

**Affiliations:** Sports Medicine Center of Fudan University, Department of Sports Medicine and Arthroscopy Surgery, Huashan Hospital, Shanghai 200040, China

## Abstract

Achilles tendinopathy is one of the most frequently ankle and foot overuse injuries, which is a clinical syndrome characterized by the combination of pain, swelling, and impaired performance. The two main categories of Achilles tendinopathy are classified according to anatomical location and broadly include insertional and noninsertional tendinopathy. The etiology of Achilles tendinopathy is multifactorial including both intrinsic and extrinsic factors. Failed healing response and degenerative changes were found in the tendon. The failed healing response includes three different and continuous stages (reactive tendinopathy, tendon disrepair, and degenerative tendinopathy). The histological studies have demonstrated an increased number of tenocytes and concentration of glycosaminoglycans in the ground substance, disorganization and fragmentation of the collagen, and neovascularization. There are variable conservative and surgical treatment options for Achilles tendinopathy. However, there has not been a gold standard of these treatments because of the controversial clinical results between various studies. In the future, new level I researches will be needed to prove the effect of these treatment options.

## 1. Introduction

The clinical symptoms of pain, swelling, and impaired physical function of Achilles tendon are common in sports and daily life. Traditionally, many terms have been used to describe the disorders, including tendinitis, tendinosis, and paratenonitis. However, recent histopathological studies have found these disorders as a result of a failed healing response, which may cause degenerative changes in the tendon. The failed healing response includes three different and continuous stages (reactive tendinopathy, tendon disrepair, and degenerative tendinopathy) [1–3]. However, inflammatory response is not found in the three stages. In 1998, Maffulli et al. suggested to use the term tendinopathy in order to describe these intratendinous disorders [4].

The two main categories of Achilles tendinopathy are classified according to anatomical location and broadly include insertional (at the calcaneus-Achilles tendon junction) and noninsertional (2 to 6 cm proximal to the insertion of the Achilles tendon into the calcaneus) tendinopathy [5].

## 2. Epidemiology

Achilles tendinopathy is one of the most frequently ankle and foot overuse injuries [6]. This disorder is more likely to be found in the individuals who participate in the physical activities such as running and jumping. It may affect 9% of recreational runners and cause up to 5% of professional athletes to end their careers [7]. In an epidemiologic investigation of 1394 nonathletes, Achilles tendinopathy was found in 5.6% of the subjects (4% insertional, 3.6% noninsertional, and 1.9% both forms) [8]. In another research, Kvist found that 20% to 25% of Achilles tendinopathy patients had insertional disorder, 66% had noninsertional, and 23% had either retrocalcaneal bursitis or insertional tendinopathy [9].

Chronic Achilles tendinopathy is more common in older people than in young people. In Kvist's study, among 470 patients who had Achilles tendinopathy, only 25% of the patients were young athletes and 10% were younger than 14 years [9]. Moreover, insertional tendinopathy tends to occur in more active persons, whereas noninsertional tendon injury tends to occur in older, less active, and overweight persons [10].

## 3. Etiology

The risk factors of Achilles tendinopathy can be divided into intrinsic and extrinsic factors, either alone or combination. Intrinsic factors include biomechanical abnormalities of the lower extremity such as leg length discrepancy hyperpronation, varus deformity of the forefoot, pes cavus and limited mobility of the subtalar joint [10], and systemic conditions such as increasing age [11, 12], inflammatory arthropathies, corticosteroid use, diabetes, hypertension, obesity, gout, hyperostotic conditions [13–16], lipidaemias, aromatase inhibitors, and quinolone antibiotics [17]. Extrinsic factors include excessive mechanical overload and training errors such as increased interval training, abrupt changes in scheduling, excessive hill training, training on hard or sloping surfaces, increased mileage, increased repetitive loading, poor shock absorption, and wedging from uneven wear [10, 15, 17].

## 4. Tendon Anatomy and Physiology

### 4.1. Histology and Anatomy

The Achilles tendon originates from the merging of the soleus muscle with the two bellies of the gastrocnemius and it is inserted distally onto the calcaneus. The normal tendon is seen as a fibrillar and generally rounded structure that is white and elastic, because most of them are avascular. Two kinds of cells, tenoblasts and tenocytes, account for 90–95% of the cellular element of the tendon. The cells in a normal Achilles tendon are well organized. The remaining 5–10% of cells are chondrocytes at the entheses and a few synovial cells in the synovial tendon sheath [18–20]. The extracellular matrix between the collagen fibers and tenocytes is composed of glycosaminoglycans, glycoproteins, and proteoglycans, whose high hydrophilicity contributes to the elasticity of the tendon [19, 21]. The tendon is mainly composed of collagen fibers, which make up 90% of the tendon protein or 70% to 80% of the dry weight of the tendon. Type I collagen is the commonest; it forms 95% of tendon collagen, and most of the collagen fibers are aligned longitudinally [22]. Other types of collagen, such as type III, type V, and type XII, are also important in the tendon [21]. Type III collagen has an important role in the healing and developing process. As type III collagen tends to produce smaller, less organized fibrils, this has implications for the mechanical strength of the tendon. Type V collagen is intercalated into the core of the type I collagen fibril, where it forms a template for fibrillogenesis and modulates fibril growth [23]. Collagen type XII has been postulated to integrate adjacent matrix components due to its ability to bind proteoglycans, fibromodulin, and decorin, while interacting with collagen type I fibrils [24]. Elastin accounts for only about 2% of the dry mass of tendon and can undergo up to 200% strain before failure [18]. Collagen forms microfibrils, fibrils, and fibers. A group of fibers constitutes a fascicle. The fascicles unite to form bundles, and they are surrounded by the endotenon, which carries blood vessels, lymphatics, and nerves [20].

The normal blood supply to the tendon is variable between different age and area. The Achilles tendon receives blood supply from three sources: the muscle-tendon junction, the bone-tendon junction, and the length of the tendon. The blood supply to middle portion of the tendon is by way of the surrounding paratenon (the most important blood supply to the tendon) [25, 26]. The most abundant blood supply zone in the tendon is at the tendon insertion, whereas, in the people who are older than 30 years, the most intensely vascularized zone is at the tendon origin. The area of the tendon approximately 2 to 6 cm above the insertion into the calcaneus is the least vascularized zone at all ages [26–29], resulting in limited reparative ability at times of stress or injury.

The nerve supply to the tendon comes from the overlying superficial nerves or from nearby deep nerves, the tibial nerve and its branches [30]. The nerves travel along with the blood vessels. Four types of afferent receptors are found either on the surface or in the tendon. The receptors are (a) Ruffini corpuscles, pressure receptor; (b) Vater-Pacinian corpuscles, movement receptor; (c) Golgi tendon organs, mechanoreceptor; and (d) free nerve endings, acting as pain receptors [26]. Golgi organs are the most abundant receptor at the tendon attachments, which can detect pressure and stretching changes in the Achilles tendon [31].

## 5. Pathology

In the Achilles tendinopathy patients, the tendon turns out to be thickened, uneven, and brownish. Histological examination of the affected tissue shows no macrophages, neutrophils, or other inflammatory cells. Therefore, traditional terms tendinitis and tendonitis are inappropriate for designating this tendon disorder [32]. As the histological studies demonstrate an increased number of tenocytes and concentration of glycosaminoglycans in the ground substance, disorganization and fragmentation of the collagen, and neovascularization, the term “tendinopathy” seems preferable. The tenocytes present at the site of degeneration have an irregular shape and a higher rate of apoptosis [10, 32–34]. These tissue changes progress to chronic mucoid and/or lipoid degeneration of the tendon with a variable amount of fibrocartilaginous metaplasia and calcium hydroxyapatite deposits [17, 35]. In chronic Achilles tendinopathy, major molecular changes include increased expression of type III collagen, fibronectin, tenascin C, aggrecan, and biglycan. These changes are consistent with repair, but they might also be an adaptive response to changes in mechanical loading because repeated minor strain is thought to be the major precipitating factor in tendinopathy [36].

Healthy tendons are relatively avascular. Neovascularization, a descriptive term for the appearance of abnormal vessels, is one feature of Achilles tendinopathy [37, 38]. Neovascularization and the accompanying neonerves have been hypothesized to be the source of pain in chronic midportion Achilles tendinopathy [3, 39–43]. Moreover, postcapillary venous filling pressures are increased at both the midportion Achilles tendon and the paratendon tissues. However, the tissue oxygen saturation in tendon does not show any difference between Achilles tendinopathy and normal tendon tissue [44]. Moreover, mechanoreceptors and nerve-related components such as glutamate N-methyl-D-aspartate (NMDA) receptors are present in association with blood vessels in tendinopathic tendons [45–47]. In a rabbit model of tendinopathy, the increased expression of angiogenesis factor (vascular endothelial growth factor (VEGF)) was detected and the pattern of vascularity showed an increase in the number of tendon blood vessels [48].

### 5.1. Natural Healing of Tendinopathy

Tendon repair involves a sequence of three phases. The first inflammatory phase lasts a few days. Erythrocytes and inflammatory cell migrate to the injury site within the first 24 h. Vasoactive and chemotactic factors are released with increased vascular permeability, initiation of angiogenesis, proliferation of tenocyte, and production of collagen fiber [49]. After a few days, the proliferative phase begins. Synthesis of type III collagen reaches a peak during this stage, which lasts for a few weeks [49]. Water content and glycosaminoglycan concentrations remain high during this stage. Tendon repair coincides with tenocyte proliferation in the epitenon and endotenon, as well as in the tendon sheath [17]. Finally, after approximately 6 weeks, the modeling stage starts. The healing tissue is resized and reshaped [49]. Syntheses of cellularity, collagen, and glycosaminoglycan are decreased. This remodeling phase starts with a fibrous consolidation process. In this period, the repair tissue changes from cellular to fibrous and the collagen fibers align along the direction of the loads applied to the tendon. Then, after the tenth postinjury week, the maturation phase occurs, with gradual change of fibrous tissue to scar-like tendon tissue over the course of one year [46].

Matrix metalloproteinases (MMPs) are a family of proteolytic enzymes, which are classified according to their substrate and primary structure. Previous studies indicated that the MMPs are important in regulating the extracellular matrix network remodeling, and their amounts are changing during Achilles tendon healing process [46].

The quality of the tissue is weakened because of the abnormal healing process, with disordered proliferation of tenocytes, degeneration change of tendon cells, disruption of collagen fibers, and eventually increasing of noncollagenous matrix. If the source of the tendon injury persists, the area of degeneration or the tear may persist or worsen over time [20].

The sources of pain in Achilles tendinopathy are very complicated. The pain may originate from multiple factors. Increased production of prostaglandins (PGs) in matrix, neovascularization in tendon body, tenocyte changes in structure and function, and metabolites changes in tendinopathy are thought to be the sources of pain [47]. Chemical irritants, including cytokines (tumour necrosis factor-alpha (TNFa) and interleukin- (IL-) 1b), signalling molecules (Ca^2+^, adenosine triphosphate (ATP)), neuropeptides (substance P (SP), neuropeptide Y), and neurotransmitters such as glutamate, have been found to be elevated in tendinopathy and proposed as causing pain [47, 48]. Recent research indicated that the nonneuronal cholinergic system also has been implicated as a factor of pain in chronic tendinopathy [49].

## 6. Treatment

### 6.1. Nonoperative Management

In the acute phase, initial rest is the most important. The use of braces and immobilization with a cast or pneumatic walking boot are combined with modified activity [10]. Immobilization is frequently used in the acute setting to control exacerbating factors, but prolonged immobilization should be avoided. Modification of training regimes and specific exercises are necessary. Most patients will be able to return to previous activities. Orthotics may be helpful alongside other modalities of treatment if there is an identifiable malalignment, whereas braces or splints do not appear to improve outcomes in Achilles tendinopathy [50]. A graduated shoe raise or heel lift can alleviate pressure on the insertion by plantar flexing the heel. Hindfoot malalignment associated with insertional disorders can be corrected by insoles, if it is thought to be a provocative factor [51].

Ultrasound is a common prescribed program of physical therapy. In animal studies, ultrasound could stimulate collagen synthesis in tendon fibroblasts and cell division during the period of rapid cell proliferation [52]. Therapeutic ultrasound has been shown to reduce the swelling in the acute inflammatory phase of soft-tissue disorders, relieve pain, and increase function in patients with chronic tendon injuries and may enhance tendon healing [53, 54]. However, due to a lack of new, high quality data on the effect of ultrasound on Achilles tendinopathy, the evidence remains insufficient to support its clinical use [55, 56].

Low level laser therapy (LLLT) could reduce the expression of proinflammatory markers such as IL-6 and TNF-*α* in gene level [57]. In the cellular level, LLLT may increase collagen production [58], stimulate tenocyte proliferation [59], downregulate MMPs, decrease the capillary flow of neovascularization, and finally preserve the resistance and elasticity of tendon [60, 61]. In a placebo-controlled, double-blind, prospective study of twenty-five patients with a total of forty-one digital flexor tendon repairs, laser therapy could reduce postoperative edema but did not relieve the pain and increase the grip strength or functional results compared with those in control group [62]. Another randomized controlled clinical study indicated that LLLT was not used in treating midportion Achilles tendinopathy [63]. A meta-analysis indicated that LLLT could potentially be effective in treatment of tendinopathy when recommended dosages were used [64]. In the future, new, high quality researches will be needed to prove the effect of LLLT in treatment of Achilles tendinopathy.

The scientific basis of NSAIDs used in chronic tendinopathy is questionable, because the histological examination in the tendinopathic tissue shows no inflammatory cells [21]. The benefits of NSAIDs use are reliving pain in the acute phase and reducing the possibility of leg stiffness [65]. However, there are some studies that indicated that the NSAIDs may inhibit tendon cell migration and proliferation and impair tendon healing [66]. Some authors believed that NSAIDs had little or no effect on the clinical outcome [5, 67].

Corticosteroid injections are reported to reduce pain and swelling and improve the ultrasound appearance of the tendon. The mechanism behind any positive effect of local steroids on chronic Achilles tendinopathy remains unclear. Some authors have hypothesized that any beneficial effects of corticosteroids in this condition are owed to other local steroid effects rather than suppression of inflammation, including lyses of tendon and peritendon adhesions or alteration of the function of pain generating nociceptor in the region [68]. Corticosteroid injections may have some benefit in the short term, but adverse effects were reported in up to 82% of corticosteroid trials [69]; these include tendon atrophy [69], tendon rupture [70], and decreased tendon strength [71, 72]. Corticosteroid injections could cause vasoconstriction through prostacyclin and adrenoceptors and inhibit nitric oxide synthase, which may be the source of adverse effects [20]. Any possible benefit of corticosteroid injection appears to be outweighed by potential risks. Overall, the evidence to support the injection of corticosteroid in or around the Achilles tendon is insufficient.

Many studies and systematic reviews have found that eccentric exercises are beneficial in the early treatment of noninsertional Achilles tendinopathy [56, 68, 73], but the mechanism of how this exercise works is poorly understood. Theoretically, the reasons of eccentric exercise in reducing pain and improving healing process include more rapid strengthening of the calf muscle, stiffening and lengthening of the myotendinous unit, and decreasing of neovascularization in the tendon [68]. The tensile force generated within the tendon during the exercise temporarily ceases blood flow through the neovessels. With repetition over time, the neovessels are obliterated, along with their associated pain receptors, which lead to the relief of symptoms [55]. The potential harms of eccentric exercise include delayed-onset muscle soreness, exacerbation of the tendinopathy, and muscle injuries [50]. This injury may increase in subjects who are unaccustomed to exercises and if sufficient recovery periods are not allowed. Muscle injuries often occur when athletes are fatigued and strength recovery may take up to 24 hours after exercise [73]. Therefore, the exercise should be taught and monitored by a health professional, such as a physiotherapist or sports medicine physician, capable of ensuring correct biomechanics and of supervising the gradual increase of tendon loading [50].

Conflicting results have been reported for extracorporeal shockwave therapy (ESWT) [20]. How ESWT works is still poorly understood, but it is known to cause selective dysfunction of sensory unmyelinated nerve fibers, alteration in the dorsal root ganglia, and cavitation in interstitial and extracellular disruption, which could promote the healing response [74]. Recently, an in vivo study found that mechanical stimulus provided by ESWT might aid the initiation of tendon regeneration in tendinopathy by promoting proinflammatory and catabolic processes that are associated with removing damaged matrix constituents [75]. In recent research, series randomized placebo-controlled trials have confirmed the high evidence of efficacy of ESWT in chronic Achilles tendinopathy [74]. However, the most effective dose and duration of ESWT are still unknown.

The introduction of platelet-rich plasma (PRP) at the site of tendon injury is thought to facilitate healing because it contains several different growth factors and other cytokines that can stimulate healing of soft tissue [55]. Animal studies indicated that PRP could increase the expression of collagen types I and III and vascular endothelial growth factor and improve the healing and remodeling process of the tendon [76, 77]. A lot of studies demonstrating improved tendon healing using PRP compared with controls, but controversial clinical results exist when using PRP to treat Achilles tendinopathy [78–84]. Ultrasound guidance allows injected PRP into the tendon with great accuracy [79, 84]. However, the evidence to support the use of PRP in the management of Achilles tendinopathy is insufficient and level I, controlled, randomized studies are still needed in the future researches.

Intratendinous hyperosmolar dextrose (prolotherapy) is thought to produce a local inflammatory response and increase in tendon strength. Clinical results indicated that intratendinous injections of hyperosmolar dextrose could reduce the pain at rest and during tendon-loading activities in patients with chronic of the Achilles tendinopathy [85, 86]. Moreover, after injection of dextrose, there were reductions in the size and severity of hypoechoic regions and intratendinous tears and improvements in neovascularity [86].

Nitric oxide is a small-free radical generated by a family of enzymes, the nitric oxide synthases. It can induce apoptosis in inflammatory cells and cause angiogenesis and vasodilation. Moreover, oxygen free radicals can stimulate fibroblast proliferation [87]. Nitric oxide can enhance tendon healing. Inhibition of nitric oxide synthase can reduce healing process, which resulted in a decreased cross-sectional area and reduced failure load [48]. Theoretical, topical glyceryl trinitrate (GTN) application could increase local tissue nitric oxide concentrations, which are believed to improve fibroblast function and wound healing [68]. Acute GTN facilitates capillary venous outflow in painful Achilles tendons. However, in another study, capillary blood-flow and tendon oxygenation remain unchanged following GTN application [88]. A randomized double-blind placebo-controlled study demonstrated improvements in symptoms using GTN patches [89]. Another randomized controlled trial found no difference between groups in scores between the two groups for pain or disability. Moreover, there has not appeared to be any histological or immunohistochemical change between groups [90].

Cryotherapy might play a role in reducing the increased capillary blood flow in Achilles tendinopathy, reducing the metabolic rate of the tendon and applying for relief of pain [91, 92]. However, recent evidence in upper limb tendinopathy indicated that the addition of ice did not offer any advantage over an exercise program consisting of eccentric and static stretching exercises [93].

The sclerosing agent that selectively targets the vascular may cause thrombosis of the vessel. As the concomitant sensory nerves have been implicated as possible pain generators, to destroy local nerves adjacent to neovessels may decrease pain [55]. As vessel number has been shown to correlate with tendon thickness, treatment that decreases vessel number is likely to also affect the tendon thickness. Moreover, the sclerosing agent injected at multiple sites around the tendon and neovessels initiates a local inflammatory response, which induces a proliferation of fibroblasts and synthesis of collagen. Therefore, a stronger, more organized tendon could be produced [52]. Early reports using sclerosing agent injected under Doppler ultrasound guidance into the abnormal vessels on the ventral aspect of the Achilles tendon demonstrated significant improvements in pain and function scores [54, 94]. However, due to the conflicting results, high quality evidence to make a recommendation for sclerosing injections is needed.

Deep friction massage (DFM) and tendon mobilization may also be helpful in the treatment of Achilles tendinopathy. DFM has been advocated for tendinopathy and paratendinopathy. Friction has been shown to increase protein output of tendon cells [95]. In combination with stretching, deep friction massage helps to restore tissue elasticity and reduce the strain in the muscle-tendon unit [52, 95]. Future randomized comparison studies are necessary to compare DFM in isolation with other modes of treatment.

Aprotinin is a broad spectrum serine protease inhibitor capable of blocking trypsin, plasmin, kallikrein, and a range of MMPs [96]. Most previous studies using Aprotinin injection in the management of Achilles tendinopathy showed a trend towards improved clinical results [97, 98]. The major potential negative of using Aprotinin is the side effect of allergy [51], but the allergic reactions can be reduced by minimizing repeat injections and recommending a delay of at least 6 weeks between injections [96].

## 7. Surgical Treatment

### 7.1. Noninsertional Achilles Tendinopathy

The goal of surgery is to resect degenerative tissue, stimulate tendon healing by means of controlled, low-grade trauma and/or augment the Achilles tendon with grafts. It has been suggested that noninvasive treatment should be tried for at least 4 months prior to operative interventions [10]. Conventional surgical treatment has consisted of open release of adhesions with or without resection of the paratenon. If >50% of the tendon has been debrided, augmentation or reconstruction is recommended [10].

Several operative treatments include percutaneous longitudinal tenotomies, minimally invasive tendon stripping, open tenosynovectomies, open debridement and tubularization, and tendon augmentation with flexor hallucis longus (FHL). Long-term results of operative interventions are promising but need more fair evidence to support the clinical use.

Complications are common in surgical procedure. In a large series of 432 consecutive patients, Paavola et al. reported wound necrosis in 3%, superficial infection in 2.5%, and sural nerve injury in 1%, with further complications including haematoma, seroma, and thrombosis. Therefore, the overall complication rate was 11% and reoperation rate was 3% [99].

### 7.2. Insertional Achilles Tendinopathy

Patients who do not respond to conservative treatment may need operative manage. No consensus exists regarding the duration before surgery, though most clinicians consider 3 to 6 months the minimum time necessary to evaluate the effect of conservative treatment [100].

The operative strategy for insertional Achilles tendinopathy is removal of the degenerative tendon and associated calcification, excision of the inflamed retrocalcaneal bursa, resection of the prominent posterior calcaneal prominence, reattachment of the insertion as required, and/or augmentation of the tendo-Achilles with a tendon transfer/graft [101, 102]. Calcaneoplasty and resection of the retrocalcaneal bursa can be performed endoscopically [102].

Biomechanical and clinical data suggest that 50% of the tendon attachment can be safely debrided without compromise of the insertion strength or risk of rerupture. When greater than 50% of the Achilles tendon is detached from the calcaneus, suture anchors are recommended to reattach the residual tendon. With extensive insertional, Achilles tendon disease and/or when greater than 75% of the tendon is excised, augmentation with local tissue, such as the flexor hallucis longus tendon and semitendinosus tendon, is advisable [102].

## 8. Conclusion

Achilles tendinopathy is a clinical syndrome characterized by the combination of pain, swelling, and impaired performance. The etiology of Achilles tendinopathy is multifactorial including intrinsic and extrinsic factors. The histological studies demonstrate an increased number of tenocytes and concentration of glycosaminoglycans in the ground substance, disorganization and fragmentation of the collagen, and neovascularization. The sources of pain in Achilles tendinopathy are very complicated. The pain may originate from multiple factors. There are variable conservative and surgical treatment options for Achilles tendinopathy. However, there is no gold standard of the treatments because of the controversial clinical results between various studies. In the future, more new level I researches are needed to prove the effect of these treatment options.
